# Assessing the Performance of Confirmatory Cognitive Tests in Screening Subjects for Early Alzheimer's Disease Clinical Trials

**DOI:** 10.1002/alz70859_106693

**Published:** 2025-12-25

**Authors:** David S. Miller, Xingmei Wang, Alan Kott

**Affiliations:** ^1^ Signant Health, Blue Bell, PA USA; ^2^ Signant Health, Prague Czech Republic

## Abstract

**Background:**

In early Alzheimer's Disease clinical trials, confirmatory cognitive tests such as the FCSRT, RBANS and Wechsler Memory Scale (WMS)‐Logical Memory II are often used at Screening to assure that only subjects with objectifiable cognitive decline are included in the clinical trial. These instruments are, however, often lengthy and challenging for both rater and subject. In this analysis of 5 large clinical trials in early AD, we tested the utility of these instruments in the screening process and assessed how much value they added on top of the MMSE inclusion criteria.

**Method:**

In addition to descriptive statistics, we used logistic regression models to test the performance of these individual instruments in relation to the screening MMSE score to exclude unsuitable subjects.

**Result:**

Of all subjects meeting MMSE related inclusion criteria (MMSE between 22 and 30), the RBANS identified 23%, the FCSRT 36% and the WMS‐LMII 37% as not eligible. All instruments identified 50% or more subjects with a MMSE score of 30 as ineligible. For those with a MMSE score of 26, the RBANS screen failed 19% of subjects, the WMS‐LMII screen failed ∼37% and the FCSRT ∼39% of subjects. With a MMSE score of 22, the RBANS screen failed 3%, the FCSRT 18% and the WMS‐LMII over 20% of subjects.

**Conclusion:**

Our analysis demonstrated the utility of confirmatory scales to screen fail subjects lacking objectifiable cognitive decline. Depending on the instrument used, between a quarter and a third of subjects were deemed not eligible. However, substantial differences in performance between the instruments used were identified, especially in potential study participants with MMSE scores of 26 or below. While the FCSRT and WMS‐LMII performed in an almost linear manner over the tested MMSE range, the RBANS performed in a curvilinear manner. Additionally, for those with MMSE scores below 25, the RBANS only identified a small subset of subjects as ineligible. Those weighing implementation of these instruments in a clinical trial should consider the presented data in the context of the MMSE inclusion criteria, subject and rater burden, and the goals of the trial.

